# Critical evaluation of applications of artificial intelligence based linguistic models in Occupational Health

**DOI:** 10.47626/1679-4435-2023-1241

**Published:** 2024-08-05

**Authors:** Mateus Lins dos Santos, Vera Nascimento Gomes Victória

**Affiliations:** 1 6ª Vara, Justiça Federal em Sergipe, Itabaiana, SE, Brazil; 2 9ª Vara, Justiça Federal em Sergipe, Propriá, SE, Brazil; 3 Comissão de Residência Médica, Fundação Beneficência Hospital de Cirurgia, Aracaju, SE, Brazil

**Keywords:** occupational medicine, artificial intelligence, natural language processing, occupational health, education, medical, medicina do trabalho, inteligência artificial, processamento de linguagem natural, saúde ocupacional, educação médica

## Abstract

This article explores the impact and potential applications of large language models in Occupational Medicine. Large language models have the ability to provide support for medical decision-making, patient screening, summarization and creation of technical, scientific, and legal documents, training and education for doctors and occupational health teams, as well as patient education, potentially leading to lower costs, reduced time expenditure, and a lower incidence of human errors. Despite promising results and a wide range of applications, large language models also have significant limitations in terms of their accuracy, the risk of generating false information, and incorrect recommendations. Various ethical aspects that have not been well elucidated by the medical and academic communities should also be considered, and the lack of regulation by government entities can create areas of legal uncertainty regarding their use in Occupational Medicine and in the legal environment. Significant future improvements can be expected in these models in the coming years, and further studies on the applications of large language models in Occupational Medicine should be encouraged.

## Abstract (pt)

Este artigo explora o impacto e as possíveis aplicações dos grandes modelos de linguagem na Medicina do Trabalho. Os grandes modelos de linguagem têm a capacidade de fornecer suporte durante a tomada de decisão médica, a triagem de pacientes, a sumarização e confecção de documentos técnicos, científicos e jurídicos, o treinamento e educação de médicos e da equipe de saúde ocupacional, bem como a educação de pacientes, potencialmente levando a menores custos, menor gasto de tempo e menor incidência de erros humanos. Apesar dos resultados promissores e da grande variabilidade de aplicações, os grandes modelos de linguagem apresentam também limitações significativas em relação à sua acurácia, ao risco de geração de informações falsas e a recomendações errôneas. Também devem ser considerados diversos aspectos éticos ainda não bem elucidados pela comunidade médica e acadêmica, e a falta de regulamentação pelas entidades governamentais pode gerar áreas de incerteza jurídica sobre o seu uso na Medicina do Trabalho e no ambiente judicial. Melhorias futuras significativas podem ser esperadas nesses modelos nos próximos anos, e mais estudos das aplicações dos grandes modelos de linguagem na Medicina do Trabalho devem ser encorajados.

## INTRODUCTION

Large language models are tools that interpret and produce pieces of text in human natural language, in a similar way to a conversation. Models such as OpenAI ChatGPT are becoming increasingly common tools in the everyday life of general population due to their ability to produce pieces of text at high speed, with a linguistic pattern similar to handwritten pieces of text and grammar error-free.

Several studies have been conducted to determine the applicability and limits of these tools in various areas of medicine, but publications aimed at occupational medicine are scarce. Until the submission of this article, no publication in the SciELO, Medline, or PubMed databases had clearly listed terms related to large natural language models and occupational health in their titles. We found only 1 article on Google Scholar, published in preprint format.^1^

### NATURAL LANGUAGE MODELS

Large natural language models are computer models capable of receiving text inputs in human language, interpreting them correctly, and providing understandable text outputs appropriate to their request. These models are trained with vast amounts of text and images, allowing for proper familiarization with various topics, delving into multiple specialties and adapting to different communication formats.^2^

However, one of the main limitations of the models available until early 2023, especially ChatGPT, was the presence of a database limited to pieces of text up to 2021, and the inability to perform internet searches and interpret images.

Currently, ChatGPT and other language models have versions that allow the model to be custom-trained based on new data, such as books, guidelines, or laws, for example. Other models, in addition to the most modern versions of ChatGPT, are also capable of searching the Internet in real time, accessing any publicly available documents. Recently, the GPT 4.0 and Bard (Google Inc.) models have also provided image interpretation, including the interpretation of scanned or handwritten pieces of text. These models are also capable of real-time conversation with humans and can be used for a wide variety of purposes.

### DECISION-MAKING SUPPORT

ChatGPT and Bard can be used to support medical decision-making in various ways. One direct way would be to use the models to answer questions through direct consultations on the desired topics, in which ChatGPT has demonstrated good technical performance. Garabet et al.^3^ showed that ChatGPT outperformed students in the first exam of the United States Medical Licensing Examination. According to Gilson et al.,^4^ ChatGPT was also able to pass tests on the AMBOSS and NBME platforms. Cai et al.^5^ found that the GPT 4.0 model obtained 71.6% correct answers in an American ophthalmology licensing exam, a result comparable to the average performance of humans, which was 72.2%, although the model performed significantly worse when interpreting questions with images.

ChatGPT can also be used to generate recommendations based on information from the clinical case involved, in which personal data on the patient, such as sex, age, complaints, and physical examination, can be provided as input to the models to request recommendations for action. Liu et al.^6^ conducted a study in which physicians had to evaluate clinical recommendations provided by ChatGPT and other specialists, and ChatGPT provided 9 of the 20 best recommendations.

However, it should be noted that the model is not close to 100% accurate and often diverges from the subject or generates erroneous information. Therefore, it should not be used as the sole source of consultation, always having the facts checked with reliable sources of medical literature.

In occupational medicine, these models can be used not only to aid decision-making in the clinical management of cases, but also to help the occupational physician identify and educate on occupational risks, including ergonomic and psychosocial risks,^7^ helping to detect risk factors for occupational diseases on a case-by-case basis and providing support for decisionmaking on sick leave, with due care when considering the model accuracy.

### SCREENING PATIENTS

Several studies have been conducted to evaluate the effectiveness of ChatGPT as a patient screening model,^8-10^ due to its ability to interact directly with patients in human language and produce easy-tounderstand answers in real time or synthesize and analyze information that can be passed on to doctors. Again, technical challenges related to the accuracy of the models and ethical ones arise with the unsupervised use of technology and occupational physicians should always consider them.

The large language models could be used as contact points available regularly, allowing occupational health personnel to contact them if they have any questions or to report any incidents that have occurred, while the model could also provide first aid guidance and signal to the occupational health team with due diligence the incident or refer the patient for a consultation at the next scheduled time, when it is not an urgent matter. Considering the current accuracy of the models and the ethical and legal risks involved, we do not recommend implementing similar models in Brazilian occupational medicine for the time being.

### DOCUMENT SUMMARIZATION

A major benefit of large language models is their capacity for interpretation and synthesis. A large, complex document can be provided to the model associated with a question of interest, and a summary with good reliability can be generated.^11^

The occupational physician can, for example, provide a Programa de Controle Médico de Saúde Ocupacional (PCMSO, Occupational Health Medical Control Program) from a company and ask the model to list which exams should be performed, according to that document, for a particular occupation or what the risks of a particular job are. It can even be questioned where the information was found in the PCMSO. This can also be done with court documents, occupational profiles, ergonomic reports, and environmental risk prevention programs, generating significant time savings and preserving the focus on the most relevant areas of these documents.

The same strategy can be applied to laws, decrees, and other technical pieces of text to guide the study or divide new themes into topics. This strategy can also be applied to internal company rules, medical records, and other documents that require medical evaluation, as long as care is taken with the privacy and security of data, and the ethical repercussions of sharing this data with the companies that produce the language models are considered.

As mentioned in previous sections, despite their great usefulness and efficiency, the accuracy of language models is also flawed for summarization tasks,^11^ so we recommend that summarization using models be used as an auxiliary tool in the occupational physician preparation and that all information be confirmed separately afterwards.

### DOCUMENT CONSTRUCTION

A key task of the great language models is the rapid production of grammatically correct pieces of text, following appropriate language rules, and with coherent content, including the possibility of controlling tone, ambience, and complexity. The occupational physician can use and exploit this ability in various spheres, from preparing medical documents, such as reports, assessments, and opinions, to producing technical documents, such as the PCMSO and scientific studies. The use of large language models can save time and effort when producing textual materials.

Although effective, several ethical and legal considerations need to be made about the production of textual materials. The immediate concern regarding the use of language models to produce pieces of text is the veracity of the information, which should always be checked for accuracy on the part of the human author of the text.

In a scientific environment, it is generally recognized that authorship does not need to be attributed to the language model used,^12^ but its use should be declared as part of the method used in the study. It is also necessary to consider that the guidelines of various journals have been updated in relation to the use of these models, with many of them restricting their use or even banning studies produced with the aid of the tool. We recommend that the authors review both the text and the publication guidelines of the chosen journal before opting to use large language models.

In the legal environment, the use of language models is still little discussed and unregulated. Until this study was submitted, they could be used in expert reports, technical opinions, or other documents with legal value. However, the author should review and take full responsibility for the content produced, regardless of the presence of errors in the language model, and should bear the consequences of these errors in the same way as they would for other types of errors of their own making.

### TRAINING AND EDUCATION

The usefulness of large language models in medical training has been the subject of extensive discussion in the literature,^2-6^ with significant potential for valid applications. As discussed previously, the synthesis capacity of the models available allows them to be used to summarize scientific and educational documents, focusing on the main points and directing learning to the areas of greatest interest.

A direct search can also be made to ask the model to generate study topics on a given subject, from which the physician can search for study materials. In addition, the model is capable of generating hypothetical clinical situations to test the physician knowledge, and ask questions based on the models of the main medical specialty exams.

The use of large language models in medical education seems promising, although the limitations of the accuracy and veracity of the information previously mentioned should be safeguarded. Therefore, we recommend fact-checking with external sources, and always asking the model to provide the sources of the information provided.

Language models can also be used to educate patients and health care personnel through userfriendly pieces of text with simplified language, available through integrations that allow them to be used on communication platforms that are common in Brazil (e.g. WhatsApp), and health care personnel can verify their content. Another possibility associated with the use of large language models is the generation of educational images and infographics using other artificial intelligence technologies, through requests to models that have this feature, such as the OpenAI DALL-E model, which should always be validated by occupational health personnel before publication.

### LIMITATIONS

Despite their multiple applications and promising performance, large language models still have important limitations to their use. The reliability of the information they provide is one of the main limitations, with technical performance far from perfect in several tests,^3,4^ and significant rates of “hallucinations,” generating false, out-of-context information. Regardless of the purpose for which the model is used, all authors should check the veracity of all material the models present before assuming it is true. It is currently understood that users are entirely responsible for all errors related to the use of these models, and could be penalized for any damage to patients or companies.

Ethical transparency should also be considered when using these models. While models do not need to be cited as authors of scientific studies or legal documents, it is prudent to mention their use in the description of the method or in appropriate statements in the body of the text. The absence of regulations on the subject in Brazilian legislation creates another legal challenge, with a significant grey area that does not allow definitive conclusions to be drawn about the application of language models in a legal environment, and the author should take responsibility for any material generated and any repercussions.

One of the most uncertain topics regarding the use of language models based on artificial intelligence is their impact on the doctor-patient relationship. Traditionally, the doctor-patient relationship is based on trust and partnership, in which both work together to achieve the best possible results for the patient health. This relationship has come under intense pressure over time, due to changes in the population culture and habits, increased access to information, economic limitations, and precarious employment relationships, making it a perennial challenge in various areas of medicine,^13^ including occupational medicine. The use of linguistic models can help occupational physicians in a variety of tasks, including education and communication with patients and workers, but these technologies should be used judiciously, avoiding the loss of empathy and dehumanization of the health care process.

When it comes specifically to occupational medicine, another major limitation arises: the enormous scarcity of studies on the subject. As mentioned above, at the time of submitting this manuscript, only 1 study had been found on the direct applicability of the major language models in occupational medicine, which was still in preprint. Further studies are needed to explore the usefulness of these models in specific applications of Brazilian occupational medicine, based on all its regional and legal particularities.

### FUTURE PROSPECTS

Many of the limitations mentioned above will provide the basis for significant advances in natural language processing technology in the coming years. The language models currently in use were launched recently, and this is an area with intense scientific production and great interest from the medical and academic community, with the likelihood of great growth in the technology over the next few years.

The accuracy and veracity of the information has been highlighted as one of the main challenges currently facing the major language models. For example, the expansion of the training database, the possibility of customized training with references and materials provided by the author and the possibility of real-time search for the model on the Internet, already available in the most recent commercial versions of ChatGPT, can significantly increase the accuracy of the models, allowing them to be used in a clinical environment and in medical education after validation, actions that could be extended in the future to the applications discussed for occupational medicine in this study.

The ethical aspects related to the use of the tool are likely to be well defined with the growing discussion in the academic community, which has reached consensus in different areas on the use of these models in the preparation of scientific studies, and clear rules for their use will probably be generated soon. Something similar can be stated about the regulation of its use by government agencies and medical organizations, which will require a solid position with the growing use of the platform.

Further future improvements that could revolutionize the technology include image interpretation, which is already being implemented in more advanced ChatGPT and Bard models, allowing, for example, qualitative analysis of medical images or photographed work environments. In addition, video interpretation could become part of the technology in the future, expanding the limits of its application.

## CONCLUSIONS

Large language models are promising tools that could have a significant impact on the practice of occupational medicine in various areas, including clinical practice, the prevention of accidents and occupational diseases, the preparation of technical and legal documents, aiding medical decision-making, medical education, and the screening and education of patients and workers. Challenges regarding the accuracy and ethical and legal aspects of using these models need to be addressed by model developers, the medical and academic communities, and regulatory bodies. Considering the great interest of the academic and medical communities in the subject, significant improvements in the technology can be expected in the future, and more studies focused on evaluating their application in occupational medicine are encouraged.

### DECLARATION OF USE OF LANGUAGE MODELS

The authors declare that they have used ChatGPT 3.5 language model for proofreading the text and assisting in the translation of the abstract, ensuring that they have checked all the content of the material after the revisions.

## References

[1] Padovan M, Cosci B, Petillo A, Nerli G, Porciatti F, Scarinci S (2023). ChatGPT in occupational medicine: A comparative study with human
experts [Internet]. MedRxiv [Preprint].

[2] Kasneci E, Sessler K, Küchemann S, Bannert M, Dementieva D, Fischer F (2023). ChatGPT for good? On opportunities and challenges of large
language models for education. Learn Individ Differ.

[3] Garabet R, Mackey BP, Cross J, Weingarten M. (2023). ChatGPT-4 performance on USMLE Step 1 questions and its
implications for medical education: A comparative study across systems and
disciplines [Internet]. Res Square [Preprint].

[4] Gilson A, Safranek CW, Huang T, Socrates V, Chi L, Andrew R (2023). How does ChatGPT perform on the United States Medical Licensing
Examination? The implications of large language models for medical education
and knowledge assessment. JMIR Med Educ.

[5] Cai LZ, Shaheen A, Jin A, Fukui R, Yi JS, Yannuzzi N (2023). Performance of generative large language models on ophthalmology
board-style questions. Am J Ophthalmol.

[6] Liu S, Wright AP, Patterson BL, Wanderer JP, Turer RW, Nelson SD (2023). Using AI-generated suggestions from ChatGPT to optimize clinical
decision support. J Am Med Inform Assoc.

[7] Uddin J, Albert A, Ovid A, Alsharef A. (2023). Leveraging ChatGPT to aid construction hazard recognition and
support safety education and training. Sustainability.

[8] Sarbay İ, Berikol GB, Özturan İU. (2023). Performance of emergency triage prediction of an open access
natural language processing based chatbot application (ChatGPT): A
preliminary, scenariobased cross-sectional study. Turk J Emerg Med.

[9] Jacob J. (2023). ChatGPT: Friend or foe?-Utility in trauma triage. Indian J Crit Care Med.

[10] Gebrael G, Sahu KK, Chigarira B, Tripathi N, Thomas VM, Sayegh N (2023). Enhancing triage efficiency and accuracy in emergency rooms for
patients with metastatic prostate cancer: A retrospective analysis of
artificial intelligence-assisted triage using ChatGPT 4.0. Cancers (Basel).

[11] Zhang H, Liu X, Zhang J. (2023). Extractive summarization via ChatGPT for faithful summary
generation [Internet]. ArXiv [Preprint].

[12] Pourhoseingholi MA, Hatamnejad MR, Solhpour A. (2023). Does chatGPT (or any other artificial intelligence language tool)
deserve to be included in authorship list?. Gastroenterol Hepatol Bed Bench.

[13] Drossman DA, Ruddy J. (2020). Improving patient-provider relationships to improve health
care. Clin Gastroenterol Hepatol.

